# Book Reviews

**Published:** 1897-07

**Authors:** 


SANMETTO IN GONORRHEA.—A bottle of Sanmetto enabled me to discharge the patient I was treating, entirely cured. Since then I have had a crop of cases of gonorrhea, such as often explodes in our midst in the form of an epidemic. In the chronic form of gonorrhea, ending in chronic cystitis and urethritis, involving the prostate gland and lymphatics, with backache, malaise, and painful micturition, I think I can say with impartiality that I know of no medicine conserving the purpose of bridging over these troubles like Sanmetto; and I know of no class of troubles which annoy physicians more. In all such cases I would say, put the patients on Sanmetto, and if they do not improve, I will give it up. Sanmetto is invaluable in such cases. J. C. ROBERTS, M. D., Pulaski, Tenn.

---

### Book Reviews.

---

DISEASES OF THE EAR, NOSE, AND THROAT, AND THEIR ACCESORY CAVITIES. By Seth Scott Bishop, M. D., L. L. D. F. A. Davis Co, Publishers.

Text-books, like medical journals, spring up in a night. There should be either marked originality in or urgent need for a text-book on any subject to warrant its advent before the eyes of the medical fraternity. Neither one of the two exists for the above publication. There are already good books on the ear, nose, and throat for the profession at large as well as more extensive publications for the benefit of the more advanced workers.

We fail to see anything markedly original in the book unless it is the remarkable prominence given to the author of the book. That, however, is not a failing confined to Dr. Bishop for this seems to be the reason for the publication of most text-books at present. The arrangement of the book follows pretty closely that of all previous text-books on the same subject. It seems to be more of a compilation from others. The book is published by the same house as is also the most excellent little work by Dr. Sajous. Had not the present work a treatise on the ear one might well imagine it to be Dr. S.'s book, since it has all the beautiful colored plates of the latter, but lacking the other commendable features. The discussion of the various topics remind one more of a quiz compend than of a text-book. The originality in the work lies in the chapter on hay fever which discusses the etiology of this affection from a uric acid standpoint, this being the author's special idea upon this obscure disease.

As was said before, the colored plates of Dr. Sajous are beautiful and of themselves are of great value. The other plates, especially the photos of the author's well-arranged offices, are well executed as also are the numerous instruments devised especially by the author. The classification of the subjects are good and the discussion of each clearly set forth. For those who are unable to get the works of Bosworth, Lennox, Browne, Sajous, Roosa, Deneh, and others this work may prove of some value.

